# Acute traumatic maculopathy

**DOI:** 10.11604/pamj.2014.18.133.4595

**Published:** 2014-06-11

**Authors:** Omar Lezrek, Mounir Lezrek

**Affiliations:** 1 Mohammed V University Souissi, Faculty of Medicine, Department A of Ophthalmology, Rabat, Morocco

**Keywords:** Maculopathy, trauma, blurry vision, Funduscopy, optical coherence tomography

## Image in medicine

A 14-year old child presented to the emergency department (ED), complaining of blurry vision one hour after he was victim of a blunt trauma of his right eye. Initially, visual acuity was Counting Fingers at 2m and 20/20 in the left eye, Slit lamp examination was normal; Funduscopy of the right eye revealed gray-white discoloration of macula and lower retina in posterior pole (A); Optical coherence tomography showed an increase in reflectivity of the inner and outer segment junction (B); 1 week later, the visual acuity improved to 20/25 in the right eye without any treatment.

**Figure 1:**
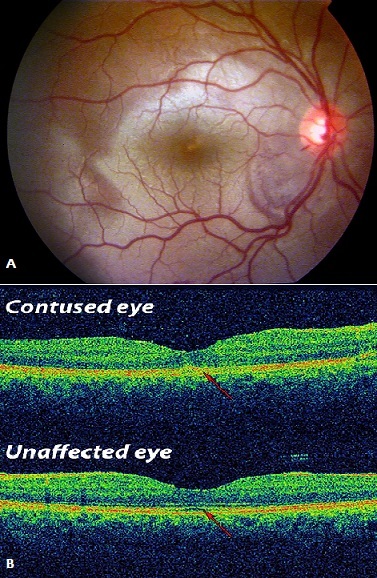
A) Fundus photography; B) difference between macular OCT in contused eye and the healthy eye

